# Supplementary material for: Effects of Co-Culture EBV-miR-BART1-3p on Proliferation and Invasion of Gastric Cancer Cells Based on Exosomes
Source: Cancers (Basel). 2023 May 19;15(10):2841. doi: 10.3390/cancers15102841 (PMC10216775; doi:10.3390/cancers15102841)
Supplement: Supplementary file 1 [file cancers-15-02841-s001.zip › cancers-2264586-supplementary.pdf]

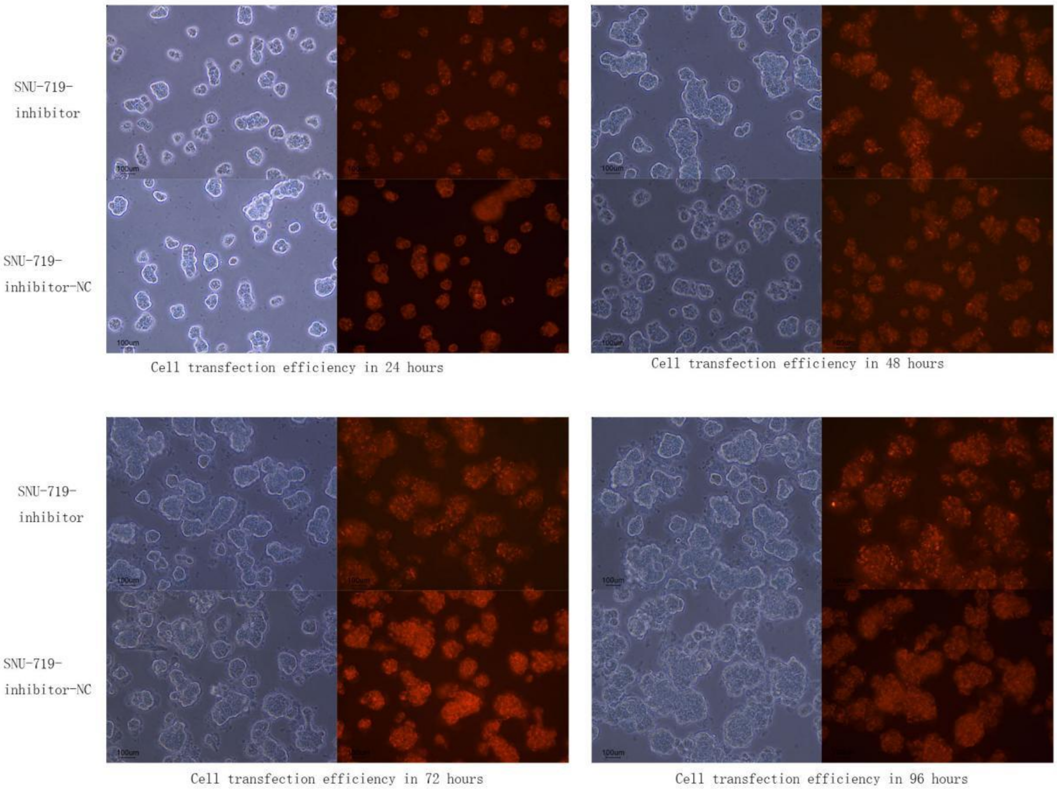

Supplementary figure 1. Cell transfection efficiency in different time.

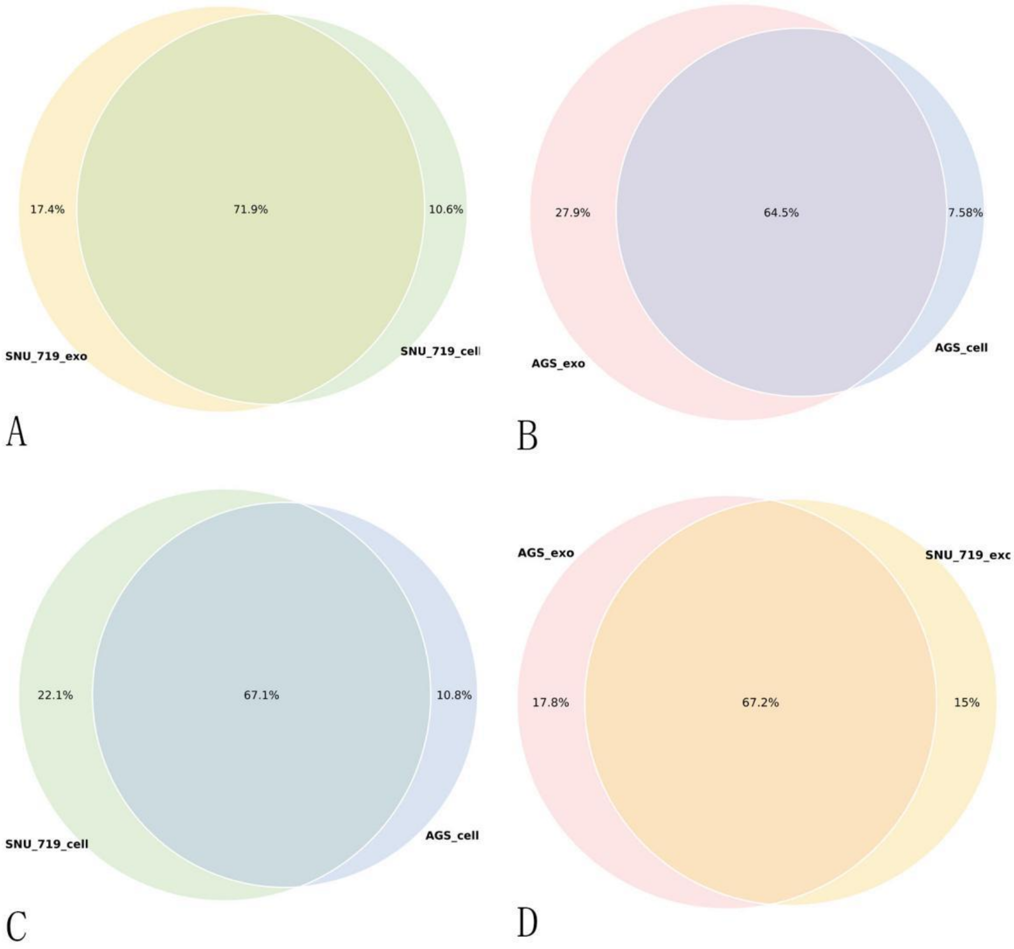

Supplementary figure 2. Distribution of small RNAs in cells and exosomes. (A-D) Venn diagrams of common and unique sequence analysis in SNU-719 and AGS cells and exosomes.
